# Annual Announcement of the Medical Department of Pennsylvania College, Session of 1848-9

**Published:** 1848-09

**Authors:** 


*Annual Announcement of the Medical Department of Pennsylvania College, Session of* 1848-9.

We are led to notice circulars from Medical Schools more fully at this time than usual, in as much as, it is to be presumed, the Medical Public are interested in knowing the position which they severally take, with respect to the measures of improvement recommended by the Medical Public through their Representatives in the National Association. In the Annual Announcement of the Medical Department of Pennsylvania College (the third Medical Institution of Philadelphia, enumerating in the order of time,) we find the following remarks on the subject of increasing the number of Professors:

"The Faculty comprises six chairs, constituting the original platform of the American schools. It is believed that among these six, all the various branches of medical science may be so distributed as to give each its due prominence, and allow the neglect of none. The natural subdivision of medical study is into six branches, three preparatory and three practical. A seventh is not an essential constituent, as is sufficiently proven by its indefinite character in many instances, the fragmentary and incoherent matters referred to it in others, and the generally prevalent confusion concerning its objects and limits. It appears to be regarded as a means of filling all and any *lacunæ* whatever in the other chairs. Such a supplementary irregularity this Faculty has not seen fit to establish. They do not, however, assert that no profitable change in the arrangement of the branches is possible. That physiology is now assuming a degree of extent and importance which renders it difficult to teach it adequately in the same course with anatomy is apparent. It will therefore require eventually, if it does not at present, a chair to itself. This addition the Faculty has had in contemplation for some time, and will perfect it so soon as they conclude that they can do so in a manner conducive to the best interests of the pupils, and without invading the essential limits and rights of the original chairs."

We submit, in the first place, that the tone of the foregoing paragraph is discourteous toward other Institutions that have adopted the recommendation to increase the number of chairs to seven. It is a fling at the latter, which might be expected in a pamphlet advocating the superlative claims of a patent nostrum, but, in our humble opinion, ill becoming a document coming from the Faculty of a Medical College. We do not hesitate thus to express freely our opinion, becanse, as we quote the paragraph to which we refer in full, the reader is enabled to form his own opinion, irrespective of any criticism of ours. In the second place, the statements so dogmatically asserted are absurdly incorrect. We would ask the Faculty of the Medical Department of Pennsylvania College to name the Institutions where the seventh chair has an "indefinite character," branches being allotted to it which are "fragmentary and incoherent," and where it is to be characterized by a "generally prevalent confusion concerning its objects and limits." Generally, we believe, in Institutions in which a seventh chair has been created, the province of instruction allotted to it is Physiology; other branches, separately or associated with Physiology, which have been selected for distinct courses are Clinical Medicine and general Pathology. Will it be contended by any Faculty that the latter subjects do not afford sufficient scope for instruction, separately from the Principles and Practice of Medicine? Is a Professor of these branches merely occupied in filling *lacunæ* in the other chairs, to quote the contemptuous figure of the circular? We trust for the credit of the Medical Department of Pennsylvania College, that they who have in charge instruction in these branches, in that Institution, as collateral to other branches, do not practically treat of them as so very insignificant as the quotation implies. But in the last part of the paragraph the Faculty say, "that Physiology is now assuming a degree of extent and importance which renders it difficult to teach it adequately in the same course with Anatomy, is apparent. It will therefore require eventually, if it does not at present, a chair to itself." What is this but a full admission that they ought, in justice to their pupils, to institute a seventh chair at once, notwithstanding the *tirade* to the contrary which immediately precedes the sentence just quoted? Would it not have been in better taste, and in a better spirit, if the Faculty had said in so many words. 'It does not suit our present convenience or interests to increase the number of our corps, but we may do so hereafter.'

With reference to dissections, the Faculty now make them essential for graduation They say:—

"In common with most of the Schools, this Institution has not hitherto made dissection an essential pre-requisite to graduation. The Faculty has resolved, however, that hereafter no candidate shall be admitted to examination who has not attended one course of Practical Anatomy. The value, and even necessity of this regulation will be apparent at once to every physician."

The Faculty express a conviction adverse to the propriety of extending the lecture session. We quote their remarks on this subject in full, and without comment:—

"It will be perceived that, while the time for commencing the course has been fixed so as to correspond with that of the other schools of the city, there has been no further increase in the length of the course. The Faculty still feel, as they always have done, convinced that the established course is long enough. All experience has proved that the pupils become wearied and exhausted by the end of that time, and that the classes grow thin before its termination. The quantity of matter presented, and the amount of attention and study required, exact from the youthful student a labor too great to be comfortably borne for a longer period. To spread the same amount of instruction over five or six months involves a long and fatal separation of the student from his private preceptor. If carried out, it will eventually destroy office tuition, or oblige the student to resort for it to the cities where the schools are located. Hitherto it has been presumed that instruction in the rudiments of the science, together with practical information, was to be expected from the private preceptor, and the fee has been proportionate. The primary relation of the pupil is to the practitioner in whose office he studies. The school is intended to supply that which the private instructor cannot give. It is not, of itself competent to give the entire amount of tuition. If the student is required to spend half the year at the school, and we then deduct the time necessary for preparation, traveling, visiting friends, &c., it is evident that the office is rendered of no account, and the name of a private preceptor can be required only *pro forma*. The true plan would be to have each student study three years with a respectable practitioner, and attend three courses of lectures, and this the Faculty of Pennsylvania Medical College urge upon their pupils. They do not and cannot require it, but they earnestly recommend it. The fees are, of course, demanded only for two sessions. Three courses of four months each, would give the student a complete year of college instruction with two of office tuition, and it is believed that this will eventually be adopted as the true plan. The Faculty dissent reluctantly from any thing that has been proposed and industriously urged before the profession as a reformatory movement, but they are convinced that all change is not necessarily reform."

